# Higher doses of a green tea-based supplement increase post-exercise blood flow following an acute resistance exercise bout in recreationally resistance-trained college-aged men

**DOI:** 10.1186/s12970-020-00358-5

**Published:** 2020-05-27

**Authors:** Carlton D. Fox, Christian T. Garner, Petey W. Mumford, Darren T. Beck, Michael D. Roberts

**Affiliations:** 1 Molecular and Applied Sciences Laboratory, School of Kinesiology, Auburn University, 301 Wire Road, Office 286, Auburn, AL 36849 USA; 2 Exercise and Performance Nutrition Laboratory, School of Health Sciences, Lindenwood University, Saint Charles, MO USA; 3 Edward Via College of Osteopathic Medicine Auburn, Auburn, AL USA

**Keywords:** Blood flow, Green tea, Resistance exercise

## Abstract

**Background:**

There are animal data suggesting green tea can enhance blood flow. However, human data are lacking. Thus, the purpose of this study was to examine the acute effects of low and high doses of a green tea-based supplement (GBS) on brachial artery blood flow before and following a resistance exercise bout.

**Methods:**

In this, double-blinded placebo-controlled trial, college-aged males (*n* = 18) who self-reported recreationally resistance training for the previous 6 ± 3 years were assigned to one of two studies including a low (300 mg serving) (*n* = 9) or high dose (600 mg serving) (*n* = 8; 1 drop) GBS study. During testing sessions, participants reported to the laboratory following an overnight fast and rested in a supine position for 15 min. Thereafter, baseline measurements for resting heart rate (HR), systolic blood pressure (SBP), diastolic blood pressure (DBP), brachial artery diameter (BAD) and blood flow (BBF) were obtained (PRE). Participants then consumed either their respective GBS dose or a similar placebo dose (microcrystalline cellulose) in a supine resting state. HR, SBP, DBP, BAD and BBF were measured 45 min after placebo or GBS ingestion (PRE2). Participants were then placed in a recumbent position and performed 4 sets of 10 arm curl repetitions using an 11 kg dumbbell. Participants returned to a supine position and HR, SBP, DBP, BAD and BBF were obtained within the first 3 min following exercise (POST), 15 min after exercise (15POST), and 45 min after exercise (45POST). Participants returned to the laboratory 24–48 h later to repeat the same protocol with either GBS or the placebo depending on randomization. Two-way (supplement x time) repeated measures ANOVAs were used to compare dependent variables between testing sessions for Study 1 (300 mg of GBS and placebo) and Study 2 (600 mg of GBS and placebo), and statistical significance was set at *p* < 0.05. No statistical comparisons were made between studies.

**Results:**

As expected, exercise increased BAD and BBF compared to resting baseline measured irrespective of supplementation. In addition, BAD and BBF did not differ between GBS and placebo at any time point after exercise in Study 1. In study 2, however, 600 mg GBS increased baseline-normalized BBF at immediately post exercise compared to placebo (placebo = 211 ± 155% increase, GBS = 349 ± 156% increase; *p* = 0.012) but not BAD.

**Conclusions:**

These data suggest a higher dose of GBS can enhance localized blood flow acutely following a resistance exercise bout. However, the long-term implications of these data are unclear, and more well-powered studies are needed to validate efficacy and elucidate potential mechanisms.

## Background

Nutritional supplements or dietary ingredients which promote vasodilation may be beneficial for increasing blood flow to working muscle during and following exercise (reviewed in [1, 2]). Common dietary supplements that have been examined in this regard have included high nitrate-containing beet root juice [3–8], L-arginine [9], L-citrulline [10–12], betalains [13], and various plant extracts (e.g., red spinach) [14]. Most of these supplements have been posited to act through increasing endothelium-dependent nitric oxide (NOx) production which, in turn, stimulates smaller artery and arteriole vasodilation. However, some supplements (e.g., betalains) appear to enhance blood flow through NOx-independent mechanisms [13]. Additionally, while numerous independent laboratories have reported beet root juice increases humoral NOx levels and vasodilation, other supplements (e.g., L-arginine and L-citrulline) have not shown consistent results (reviewed in [2]).

While less-studied in humans, there is a body of evidence suggesting green tea catechins may also act as a putative blood flow-enhancing supplement. For instance, Potenza et al. [15] reported epigallocatechin gallate (EGCG) stimulates the formation of NOx and reduces blood pressure in spontaneously hypertensive rats, and similar results have been reported in other rodent studies [16, 17]. However, unlike several of the aforementioned supplements, the effects of green tea supplements on peri-exercise blood flow patterns in humans have not been examined. Although the enhancement of peri-exercise blood flow does not have strong evidence to support ergogenecity, there is evidence to suggest enhanced blood flow supports certain phenotypic outcomes. For instance, older individuals with enhanced microvasculature properties may experience greater muscle growth during periods of resistance training [18]. Likewise, others have shown that an age-associated impairment in endothelial function reduces the anabolic response to amino acid feeding [19]. While these data are limited to older populations, these findings warrant future research into whether the enhancement of peri-exercise blood flow in proximity to a resistance training bout may provide certain anabolic benefits. There is also a plethora of literature to support the use of “blood flow enhancing” supplements to increase work and power output during submaximal and maximal exercise efforts. While mechanisms are currently lacking, it seems that enhanced blood flow through these supplements act to improve exercise economy (i.e., the amount of work able to be performed divided by oxygen consumption) (reviewed in [1, 20]. Thus, there is an interest in determining whether nutritional supplements augment peri-exercise blood flow.

The purpose of this study was to determine if lower (300 mg serving) or higher doses (600 mg serving) of a green tea-based supplement (GBS) affects brachial artery blood flow in college-aged males prior to and following acute resistance exercise compared to a placebo supplement. Given the previously mentioned animal studies, we hypothesized that lower and higher doses of GBS would enhance blood flow prior to and following exercise versus a placebo comparator.

## Methods

### Participants

This randomized double-blinded placebo controlled investigation was approved by the Institutional Review Board at Auburn University (Protocol#19–162 AR 1905). Participants were included if they were recreationally active resistance trainers and free of cardio-metabolic diseases (e.g., morbid obesity, type II diabetes, severe hypertension) or conditions which precluded exercise participation. Additionally, participants could not have consumed green tea-containing supplements, or other putative blood flow-enhancing supplements within one-month prior to participation. All participants provided verbal and written consent to participate, and this study conformed to the standards set by the latest revision of the Declaration of Helsinki.

### Experimental design

In order to assess the efficacy of GBS on peri-exercise blood flow, we opted to examine two doses (300 mg and 600 mg); for the sake of clarity, we have termed these aims as “Study 1” and “Study 2” here in the methods. The first 9 participants that enrolled in the study were placed in Study 1 which examined 300 mg of GBS (Vaso6, Compound Solutions, Carlsbad, CA, USA) versus 300 mg placebo (microcrystalline cellulose, Compound Solutions). The remaining participants were placed in Study 2 which examined 600 mg of GBS versus 600 mg placebo.

All participants completed two workout visits which are described in greater detail below. During visit #1 in both Study 1 and Study 2, participants were randomized to consume GBS or placebo using a coin flip method where one side of the coin indicated participants were to consume “A” pills, and the other side of the coin participants were to consume “B” pills. After completion of the first testing session, participants were required to return to the laboratory within 24–48 h for a second testing session to repeat these tests with either the placebo or GBS depending the coin flip randomization mentioned above. Critically, both the investigators and participants were blinded regarding which supplement as “A” versus “B”. Likewise, supplements were in pill form and were undistinguishable to all parties involved.

The remainder of the methods describes the testing sessions as well as the experimental procedures used throughout the study.

### Testing sessions

To control for the effects of diet and circadian patterns that affect hemodynamics, all participants reported to testing sessions during the morning hours (0700–1000) following an overnight fast. Additionally, participants were asked participants to refrain from all forms of structured resistance or endurance exercise the day prior to visit #1 as well as between visits #1 and #2.

We adopted a study design similar to one that our laboratory has used in the past to examine the blood flow-enhancing efficacy of an L-citrulline-containing nutritional supplement [10]. The main difference between studies is that we previously employed a leg extensor exercise bout and examined femoral artery blood flow patterns, whereas herein participants performed arm curl exercises and we examined brachial artery blood flow patterns. Notably, lower-body work with ultrasound has technical challenges given that: a) assessing the femoral artery can be intrusive with probe placement, and b) if calf extensions were allocated, consistently probing the popliteal artery is a technical challenge. Conversely, the brachial artery is relatively easy to probe, and the vessel is responsive to upper body exercise. Thus, this approach was adopted for the current study. Specific testing procedures are described in greater detail below.

#### Bioelectrical impedance spectroscopy for whole-body composition assessment (first visit only)

Body mass and composition was measured by bioelectrical impedance spectroscopy (BIS) using the SOZO device (ImpediMed Limited, Queensland, AU) according to the methods described by Moon et al. [21]. These methods have been shown to produce test-retest intraclass correlation coefficients (ICC) > 0.990 for whole body intracellular and extracellular water metrics on 24 participants [22], and these devices estimate body fat percentage based on these metrics. Notably, body composition assessments were only used for phenotyping purposes, and were not a primary outcome measure.

Following body composition testing, participants were positioned on an athletic training table in a supine position for 15 min to allow for resting hemodynamic measures to be obtained. Thereafter, resting heart rate, systolic blood pressure (SBP), diastolic blood pressure (DBP), brachial artery diameter and blood flow were obtained as pre measures (PRE). Participants consumed either 300 mg of GBS or placebo with 50 mL of tap water (Study 1, *n* = 9), or 600 mg of GBS or placebo with 50 mL of tap water (Study 2, *n* = 9 with 1 drop).

Heart rate, SBP, DBP, brachial artery diameter and blood flow were obtained 45 min following supplement ingestion (PRE2). Participants were placed in a recumbent position and performed 4 sets of 10 arm curl repetitions using an 11 kg dumbbell. Participants then returned to a supine position and heart rate, SBP, DBP, brachial artery diameter and blood flow were obtained within a 3-min post exercise window (POST), 15 min following exercise (15POST), and 45 min following exercise (45POST).

Testing procedures for each of these measures are described in further detail below.

#### Ultrasound for brachial artery blood flow assessment

Blood flow through the right brachial artery was assessed using high resolution ultrasound (Logiq S7 R2 Expert; General Electric, Fairfield, CT, USA) with a 3 to 12 MHz multi-frequency linear phase array transducer. The brachial artery was imaged longitudinally 8–10 cm distal from the shoulder joint. Simultaneous measurement of artery diameter and blood velocity was performed using duplex mode imaging (B-mode and Doppler) and video was captured through a digital interface at 30 frames/s with real time analysis (FMD Studio, Pisa, Italy). Measurements were captured for 2 continuous minutes with the transducer held by a supporting device in the same position. Vessel diameters were determined frame-by-frame via automatic edge detection software (FMD Studio, Pisa, Italy) measuring the distance between the near and far wall of the intima. Blood velocity was determined via selection of a region of interest around the Doppler waveform and a trace of the velocity-time integral was used to calculate mean velocity for each cardiac cycle. Blood flow from continuous diameter and mean blood velocity measurements during ultrasonography were calculated as [Π * (diameter/2)^2^ * time average mean velocity * 120 s].

#### Heart rate and blood pressure determination

Heart rate and blood pressure determinations occurred on the left arm simultaneously as right arm brachial artery blood flow data was being obtained using an automated blood pressure cuff (OMRON BP785, Omron Corporation, Kyoto, Japan). Triplicate values were averaged to obtain final values. The average time needed to obtain triplicate values was approximately 60–90 s.

#### Acute arm curl exercise bout

Following PRE2 assessments, all participants were seated in a chair and performed 4 sets of 10 repetitions using a 25-pound (11 kg) dumbbell. Participants were allowed 3 min of recovery between sets, and participants returned to the athletic training table and reclined in a supine position immediately following the fourth set for POST, 15POST and 45POST assessments. Given that the POST assessments occurred in closest proximity to exercise, a brief description will be provided on that time point. In short, it took approximately 8–10 s for participants to return to the athletic training table in a supine position. During the first minute following exercise, blood pressure and heart rate assessments began on the left arm, and triplicate measurements were completed by the second minute following exercise. The right arm brachial artery was located within the first minute following exercise, and data were obtained 1–3 min post-exercise. Thus, while these variables are described as “POST” throughout, the reader should be aware of the study mechanics described here.

### Statistics

Two-way (supplement x time) repeated measures ANOVAs were used to compare dependent variables between testing sessions for Study 1 (300 mg of GBS and placebo) and Study 2 (600 mg of GBS and placebo), and statistical significance was set at *p* < 0.05. No statistical comparisons were made between studies. In cases where Mauchly’s test of sphericity indicated that data were not normally-distributed over time, Huynh-Feldt correction factors were applied to the data. LSD post hoc testing was performed when a significant supplement x time interaction was observed to determine which time points differed for a dependent variable between the placebo and GBS conditions. All data herein are presented in figures and tables as means ± standard deviation values, and raw data are available in Supplemental File 1.

## Results

### Participant characteristics

Participant characteristics are presented in Table 1. Although 18 participants completed the protocol, one participant was dropped from the analysis because his blood flow response to exercise was ~ 2-fold greater than all other participants, and this response was > 3 standard deviations from the mean response. Of the remaining 17 participants, all but one participant (16 of 17) self-reported being recreationally resistance-trained 6 ± 4 h per week for 6 ± 3 years.

**Table 1:** Participant characteristics

Variable	Mean ± SD
*Low GBS dose (300 mg) experiment (n = 9)*	
Age (years)	24 ± 4
Body mass (kg)	83.0 ± 9.4
Body fat (%)	22.6 ± 5.6
*High GBS dose (600 mg) experiment (n = 8)*	
Age (years)	26 ± 4
Body mass (kg)	82.7 ± 10.0
Body fat (%)	18.1 ± 3.8

Legend: All data are presented as means ± standard deviation (SD) values

### Heart rate and blood pressure responses

Heart rate and blood pressure responses to the low and high dose GBS experiments are presented in Table 2. In the lower-dose experiment there was significant time effect for heart rate (*p* = 0.005), and post hoc testing indicated that this variable significantly decreased at PRE2 and 45POST relative to PRE (*p* < 0.05). There was also a significant time effect for SBP (*p* = 0.001), and post hoc testing indicated that this variable significantly increased at POST relative to PRE (*p* < 0.05). However, there were no supplement x time effects for any of these variables.

**Table 2:** Heart rate and blood pressure responses

Time point	PRE	PRE2	POST	15POST	45POST	Statistics
*Low GBS dose (300 mg) (n = 9)*						
Heart rate (bpm)		#			#	Supp *p* = 0.702
GBS	62 ± 12	59 ± 10	60 ± 10	62 ± 12	60 ± 10	**Time*p*** **= 0.005**
Placebo	61 ± 13	57 ± 12	60 ± 11	58 ± 11	58 ± 11	SxT *p* = 0.669
SBP (mmHg)			*			Supp *p* = 0.924
GBS	115 ± 8	115 ± 7	119 ± 9	114 ± 9	114 ± 8	**Time*p*** **= 0.001**
Placebo	115 ± 8	114 ± 7	120 ± 7	113 ± 5	116 ± 7	SxT *p* = 0.867
DBP (mmHg)						Supp *p* = 0.516
GBS	64 ± 7	63 ± 7	63 ± 7	63 ± 6	62 ± 6	Time *p* = 0.939
Placebo	63 ± 6	65 ± 6	65 ± 5	64 ± 6	66 ± 6	SxT *p* = 0.475
*High GBS dose (600 mg) (n = 8)*						
Heart rate (bpm)					#	Supp *p* = 0.825
GBS	56 ± 8	55 ± 10	56 ± 11	55 ± 9	54 ± 7	**Time*p*** **= 0.027**
Placebo	56 ± 8	54 ± 9	56 ± 8	56 ± 8	54 ± 8	SxT *p* = 0.473
SBP (mmHg)			*			Supp *p* = 0.341
GBS	112 ± 6	113 ± 6	120 ± 7	113 ± 7	114 ± 7	**Time*p*** **< 0.001**
Placebo	116 ± 4	114 ± 4	120 ± 4	116 ± 4	115 ± 3	SxT *p* = 0.239
DBP (mmHg)						Supp *p* = 0.857
GBS	64 ± 5	66 ± 5	67 ± 5	67 ± 6	68 ± 6	Time *p* = 0.454
Placebo	67 ± 7	66 ± 7	66 ± 5	67 ± 4	66 ± 6	SxT *p* = 0.397

Legend: All hemodynamic data are presented as mean ± SD. Abbreviations: *bpm* Beats per minute, *mmHg* Millimeters of mercury, *SBP* Systolic blood pressure, *DBP* Diastolic blood pressure, *PRE* Pre-supplement consumption; *PRE2* 45-min post-supplement consumption, *POST* within 3 min of arm curl exercise (4 sets of 10 repetitions using 11 kg), *15POST* 15 min post-exercise, *45POST* 45 min post-exercise, *Supp* main effect of supplement (GBS versus placebo), *Time* main effect of time, *SxT* supplement by time interaction. Symbols: *, time effect post hoc indicated greater than PRE (*p* < 0.05); #, time effect post hoc indicated lower than PRE (*p* < 0.05)

In the higher-dose experiment there was significant time effect for heart rate (*p* = 0.027), and post hoc testing indicated that this variable significantly decreased at 45POST relative to PRE (*p* < 0.05). There was also a significant time effect for SBP (*p* < 0.001), and post hoc testing indicated that this variable significantly increased at POST relative to PRE (*p* < 0.05). However, there were no supplement x time effects for any of these variables.

### Blood flow and brachial artery diameter responses

**Fig. 1:**
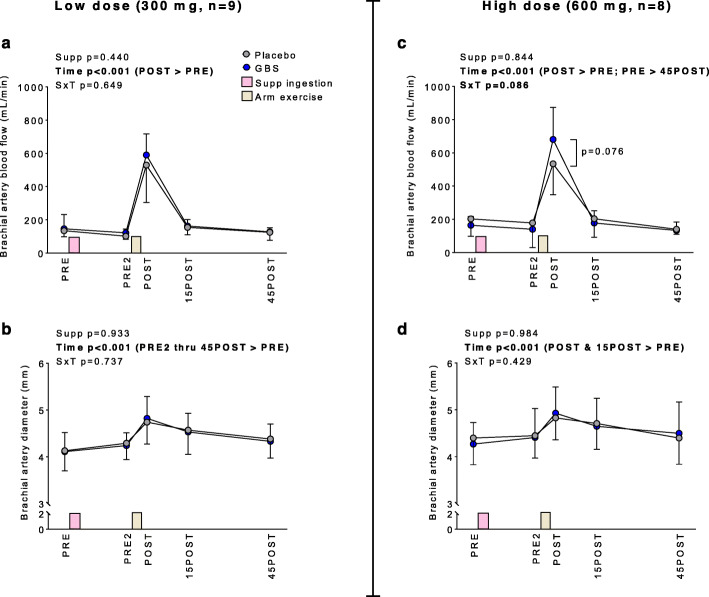
Blood flow and brachial artery responses to lower and higher doses of GBS. Legend: The effects of lower and higher doses of GBS on brachial artery blood flow (panels **a** and **c**, respectively) and brachial artery diameter (panels **b** and **d** respectively). All data are presented as means±SD values. Abbreviations: PRE, pre-supplement consumption; PRE2, 45-min post-supplement consumption; POST, within 5 min of arm curl exercise (4 sets of 10 repetitions using 11 kg); 15POST, 15 min post-exercise; 45POST, 45 min post-exercise; Supp, main effect of supplement (GBS versus placebo); Time, main effect of time; SxT, supplement by time interaction

Blood flow and brachial artery diameter responses to the lower- and higher-dose GBS experiments are presented in Fig. 1. In the lower-dose experiment there were significant time effects for blood flow (*p* < 0.001) (Fig. 1a) and brachial artery diameter (*p* < 0.001) (Fig. 1b). Post hoc testing indicated that blood flow significantly increased at POST relative to PRE (*p* < 0.001), and diameter increased at PRE2 thru 45POST relative to PRE (*p* < 0.05). However, there were no supplement x time effects for these variables.

In the higher-dose experiment there was a significant time effect for blood flow (*p* = 0.001) (Fig. 1c). Post hoc testing indicated that blood flow significantly increased at POST relative to PRE (*p* < 0.001) and 45POST was lower than PRE (*p* < 0.05). The supplement x time interaction also approached significance (*p* = 0.086), and post hoc testing indicated GBS trended higher than placebo at POST (*p* = 0.076).

There was a significant time effect for brachial artery diameter (*p* < 0.001) (Fig. 1d). Post hoc testing indicated diameter increased at POST and 15POST relative to PRE (*p* < 0.05). However, there were no supplement x time effects for this variable.

**Fig. 2:**
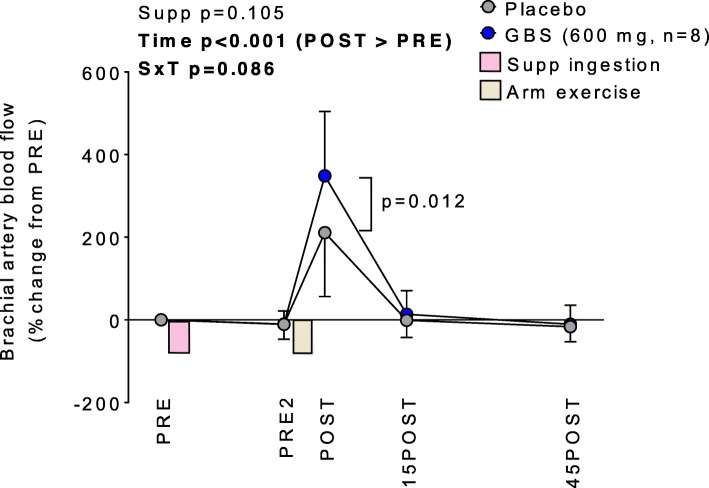
Blood flow normalized to PRE values during the higher GBS dosage experiment. Legend: The effects of higher doses of GBS on brachial artery blood flow normalized to PRE values. Data are presented as means±SD values. Abbreviations: PRE, pre-supplement consumption; PRE2, 45-min post-supplement consumption; POST, within 5 min of arm curl exercise (4 sets of 10 repetitions using 11 kg); 15POST, 15 min post-exercise; 45POST, 45 min post-exercise; Supp, main effect of supplement (GBS versus placebo); Time, main effect of time; SxT, supplement by time interaction

Given the blood flow trend for the higher dose GBS experiment, we normalized blood flow as percent change from the PRE testing point value in a post hoc fashion in order to derive blood flow changes relative to pre-supplement ingestion (Fig. 2). Notably, this approach has been used prior in order to reduce the between-subject variability in baseline blood flow measurements prior to an experimental treatment [23]. Again, the supplement x time interaction approached significance (*p* = 0.086), and post hoc testing indicated 600 mg GBS was significantly greater than 600 mg placebo at POST (*p* = 0.012).

## Discussion

There is ample evidence suggesting select nutritional supplements are capable of enhancing peri-exercise blood flow and, as mentioned prior, numerous rodent studies have reported that green tea catechins are capable of promoting vasodilation [15–17]. However, this is the first study demonstrating that a green tea-based supplement can enhance localized blood flow in humans following a resistance exercise bout.

While these data are promising, there are still several questions that remain to be answered. First, it is unclear why 600 mg GBS was effective at increasing post-exercise blood flow, whereas 300 mg was not. While human data are lacking, it is notable that researchers have shown EGCG to have a dose-dependent effect on rodent hind limb microvascular vasodilation [24]. Thus, our data imply that (on average) a 600 mg dose may be efficacious at eliciting an increase in post-exercise blood flow in humans, whereas lower doses are not as effective. It is also curious that superior post-exercise blood flow increases during the 600 mg GBS versus placebo condition were observed without an increase in brachial artery diameter. Physiological variables that affect blood flow include vessel diameter, vessel length, blood viscosity, heart rate, and blood pressure [25]. It is logical to assume that the acute ingestion of GBS did not affect vessel length and likely did not affect blood hematocrit levels or blood viscosity, although we did not assay for the latter two variables. We also posit that GBS-induced increases in blood flow were not due to increased heart rate or blood pressure given that these readings were similar between the 600 mg GBS and placebo conditions. However, it is possible that higher-dose GBS ingestion enhanced the vasodilation of smaller arterioles downstream of the brachial artery which, in turn, decreased peripheral resistance to blood flow allowing for increased velocities. Given that we currently lack these data, future studies implementing the current study design and using small vessel monitoring techniques (e.g., NIRS [26] or stain-gauge plethysmography [27]) are needed to validate this hypothesis. It is also notable that humoral signaling mediators (e.g., prostaglandins) as well as localized factors (e.g., smooth muscle NOx and potassium concentrations) affect blood flow [28]. Thus, it is possible that higher doses of GBS may operate through these mediators, and more research is needed in this regard.

Although 600 mg of GBS transiently increased post-exercise blood flow, it is currently unclear how this phenomena would affect resistance training adaptations. As mentioned prior, recent research in older populations has suggested peri-exercise blood flow may positively impact the anabolic response to feeding and resistance training. Critically, the current study does not guarantee or even insinuate that GBS supplementation during resistance training would enhance muscle mass gains. In fact, the nutritional supplement arginine, which is known to stimulate vasodilation, has been shown not to affect muscle mass changes in men that resistance trained [29]. Notwithstanding, arginine and other supplements that increase per-exercise blood flow (e.g., L-citrulline and beet root juice and extracts) have been found to prolong work and/or power output during maximal resistance exercise repetition attempts, sprinting efforts, and submaximal aerobic exercise events [10, 12, 13, 29–33]. Therefore, future research should be performed to determine if the GBS-induced increase in peri-exercise blood flow is associated with any of these potential ergogenic effects.

There are certain limitations to this study. First, we lack mechanistic data as mentioned above, and it is currently unclear why 600 mg GBS did not affect blood flow prior to exercise. Indeed, we have observed a similar phenomenon with a supplement containing L-citrulline. Specifically, compared to a placebo condition, femoral artery blood flow was not affected up to 45 min following supplement ingestion and prior to a leg extensor resistance training bout, but was significantly greater immediately post-exercise [10]. Unlike nitrate-containing supplements, it is possible that some “blood-flow enhancing” nutritional supplements may act, in part, to potentiate localized factors released from skeletal muscle or endothelial cells to enhance blood flow. To this end, more mechanistic data are needed to validate this hypothesis. This study was also limited to n-sizes of 8–9 participants in the lower- and higher-dose studies due to resource constraints. Hence, more well-powered studies (i.e., ~ 20–30 participants with a cross-over design based upon sample size calculations using the current data) are needed to validate the efficacy of GBS, and elucidate potential mechanisms through which blood flow is enhanced. Additionally, it is currently unknown if higher doses (e.g., 900 or 1200 mg) have additive effects on enhancing blood flow and/or brachial artery diameters, and this warrants further consideration. Finally, it is notable that only a standardized dumbbell (11 kg) was used for the exercise bout for all participants. Our reasoning for this was that, unlike other exercises such as the leg press or back squat, our laboratory has found it difficult in the past to assess a one-repetition maximum using a single-arm curl exercise. Thus, we were against attempting to prescribe the arm curl exercise bout based upon a predetermined relative strength, and instead posited that 11 kg is a weight that is commonly used by recreational lifters for higher-volume sets in the gymnasium.

## Conclusions

This is the first evidence suggesting a green tea-based supplement enhances localized blood flow following a resistance exercise bout. Indeed, more definitive evidence is needed to validate mechanisms, and the long-term implications of these findings for individuals who habitually resistance train are unknown and require further investigation.

## Supplementary information

**Additional file 1.**

## Data Availability

All raw data are available in Supplemental File 1.

## Abbreviations

BAD: Brachial artery diameter
BBF: Brachial artery blood flow
DBP: Diastolic blood pressure
HR: Heart rate
SBP: Systolic blood pressure
